# The Neurobiology of Personal Control During Reward Learning and Its Relationship to Mood

**DOI:** 10.1016/j.bpsc.2018.09.015

**Published:** 2019-02

**Authors:** Liana Romaniuk, Anca-Larisa Sandu, Gordon D. Waiter, Christopher J. McNeil, Shen Xueyi, Matthew A. Harris, Jennifer A. Macfarlane, Stephen M. Lawrie, Ian J. Deary, Alison D. Murray, Mauricio R. Delgado, J. Douglas Steele, Andrew M. McIntosh, Heather C. Whalley

**Affiliations:** a Division of Psychiatry, University of Edinburgh, Edinburgh, United Kingdom; b Centre for Cognitive Ageing and Cognitive Epidemiology, Department of Psychology, University of Edinburgh, Edinburgh, United Kingdom; c Aberdeen Biomedical Imaging Centre, Institute of Medical Sciences, University of Aberdeen, Aberdeen, United Kingdom; d Behaviorial Neuroscience, School of Medicine, University of Dundee, Dundee, United Kingdom; e Department of Psychology, Rutgers University, Newark, New Jersey

**Keywords:** Depression, Imaging, Locus of causality, Perceived control, Reward learning, Value of choice

## Abstract

**Background:**

The majority of reward learning neuroimaging studies have not focused on the motivational aspects of behavior, such as the inherent value placed on choice itself. The experience and affective value of personal control may have particular relevance for psychiatric disorders, including depression.

**Methods:**

We adapted a functional magnetic resonance imaging reward task that probed the value placed on exerting control over one’s decisions, termed choice value, in 122 healthy participants. We examined activation associated with choice value; personally chosen versus passively received rewards; and reinforcement learning metrics, such as prediction error. Relationships were tested between measures of motivational orientation (categorized as autonomy, control, and impersonal) and subclinical depressive symptoms.

**Results:**

Anticipating personal choice activated left insula, cingulate, right inferior frontal cortex, and ventral striatum (*p*_*familywise error–corrected*_ < .05). Ventral striatal activations to choice were diminished in participants with subclinical depressive symptoms. Personally chosen rewards were associated with greater activation of the insula and inferior frontal gyrus, cingulate cortex, hippocampus, thalamus, and substantia nigra compared with rewards that were passively received. In participants who felt they had little control over their own behavior (impersonal orientation), prediction error signals in nucleus accumbens were stronger during passive trials.

**Conclusions:**

Previous findings regarding personal choice have been verified and advanced through the use of both reinforcement learning models and correlations with psychopathology. Personal choice has an impact on the extended reward network, potentially allowing these clinically important areas to be addressed in ways more relevant to personality styles, self-esteem, and symptoms such as motivational anhedonia.

SEE COMMENTARY ON PAGE 105

Disruptions in motivation and reward processing are key elements of many psychiatric disorders, including anhedonia in major depressive disorder (MDD), negative symptoms in schizophrenia, and mania in bipolar disorder (1). Standard reward tasks (e.g., monetary incentive delay) allow examination of reward prediction, anticipation, and consumption [for review see 2, 3]. However, it has become apparent that reward processing is affected by whether an individual values being able to make his or her own choices—the inherent value of exercising personal control 4, 5. Being able to exert control over one’s own environment is beneficial to psychological well-being (6), making investigations of such concepts relevant for patient groups and the wider population. Indeed, self-determination theory (7) argues that our core needs are for autonomy (experience of enacting personal volition), competence (sense of mastery over one’s environment), and relatedness (social belonging). These determine inclination to pursue behavior for its own intrinsic enjoyment (8), which is at the heart of motivational anhedonia.

Derived from self-determination theory is the concept of the locus of causality, describing the source from which a person perceives his or her behaviors to be motivated (9): 1) Autonomy-oriented individuals are intrinsically self-motivated, seeking out opportunities for information gathering, personal challenge, and self-determination. 2) Control-oriented individuals take cues from environmental factors, e.g., reward, deadlines, and public opinion. 3) Impersonal-oriented individuals feel they have little intentional control over their behavior, deferring to concepts such as luck or fate. Notably, the impersonal orientation has previously been associated with depressive symptoms within healthy individuals (9).

Factor analysis suggests that the causality orientations have partial overlap with personality concepts described by the NEO Five-Factor Inventory: Control shares variance with agreeableness, and impersonal shares variance with neuroticism, whereas autonomy stands as a separate entity (10). Moreover, whereas traits such as neuroticism are relatively stable over the life span and have a significant genetic underpinning (11), one’s locus of causality is considered more dynamic (12) and is likely more environmentally adaptive.

Neurobiologically, the feeling of personal control (13), even when illusory (14), is associated with striatal activation, which suggests it may itself incur an additional value signal not typically captured by reward-learning paradigms. Leotti and Delgado 15, 16 attempted to isolate this within a reward learning context by testing whether the mere anticipation of control, elicited by a cue signaling an opportunity to make a choice versus a passive selection, would recruit neural systems of reward. They found that cues indicating personal control elicited greater reward system activation in both reward-obtaining (15) and loss-avoiding (16) contexts. However, this previous paradigm did not clearly dissociate between choice anticipation and receipt of the reward itself. In the present study, we have adapted this value of choice task to clearly separate anticipation and outcome phases of choice and applied reinforcement learning models to better characterize the relationship between the value of choice and neural activation in healthy individuals.

Specifically, our aims were to 1) verify previous findings concerning choice-anticipatory activation; 2) determine if responses to rewards differ according to whether or not they were personally won or passively received; 3) establish that, with appropriate modification of the original paradigm, computational models of reinforcement learning can explain observed brain activity; and 4) determine whether elicited activation covaries with subclinical depressive symptoms and personality factors relevant to depression, namely, neuroticism and measures of causality orientation. We anticipated that high neuroticism and impersonal scores would be associated with diminished activation to the inherent value of choice because depression has been linked to other types of blunted reward value (17). We were particularly interested in the roles that the striatum and dopaminergic midbrain may play, given their key importance in reinforcement learning, incentive salience, and hedonic signaling.

## Methods and Materials

### Participants

Individuals were selected from a wider ongoing study [Stratifying Resilience and Depression Longitudinally (18)] and underwent lifetime diagnostic screening using the Structured Clinical Interview for DSM-IV-TR Axis I Disorders (19) and DSM-IV-TR criteria. Only individuals without a lifetime diagnosis of major mental illness were included in the current analyses, which were performed when data from the first 149 healthy control participants were available. The following people were excluded: 15 people owing to nonperformance of the task (no response or incorrect for >33% of trials), 6 people owing to scan acquisition technical difficulties, and 6 people owing to excessive motion (more than three events involving motion greater than [0.5 × largest voxel dimension = 2.5 mm]). After these exclusions, there were 122 participants. All participants provided written informed consent, and the study was approved by local and regional ethics committees.

### Neuropsychology and Behavioral Analyses

Neuropsychological data collected included the General Causality Orientations Scale (20), which examines the sources from which a person is motivated to act (9) and consists of three dimensions: autonomy, control, and impersonal. Neuroticism scores, the severity of depressive symptoms, and handedness were also assessed (see Supplement).

### Neuroimaging Data Acquisition and Preprocessing

Data were acquired using a 3T magnetic resonance imaging scanner (repetition time = 1.56 seconds) (see Supplement).

### Modified Inherent Value of Choice Imaging Task

**Figure 1:**
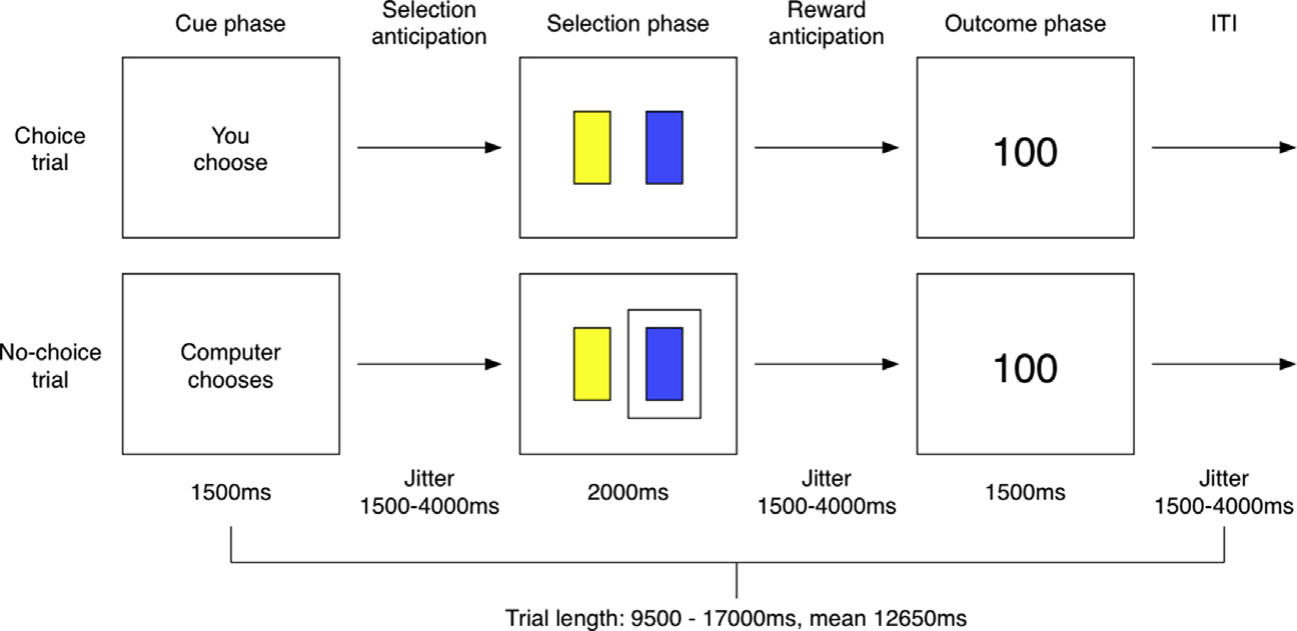
Modified inherent value of choice task. ITI, intertrial interval.

The task was adapted from Leotti and Delgado (15) and implemented in NeuroBehavioural Systems Presentation software (NeuroBehavioural Systems Presentation, Inc., Berkeley, CA). Each trial had three phases (Figure 1): 1) the cue phase, where participants learned whether they would personally be making the reward decision (choice value trial) or would be following the computer’s direction (no-choice value trial); 2) the selection phase, whereby a decision was made between a yellow or blue card; and 3) the outcome phase, when participants received a probabilistic reward according to their decision. During the selection phase, participants were able to freely select their preferred card during choice trials; on no-choice trials, a rectangle appeared around the card that the computer had selected for them, which they were obliged to confirm. Selections were made via a button press.

In the original task, the yellow and blue cards shared equal reward contingencies. In our adaptation, they had different contingencies to permit modeling of reinforcement learning: the yellow card was associated with an 80% chance of a 100-point reward, and the blue card was associated with a 20% chance. The alternative outcome was 0 points. We also introduced 1500 to 4000 ms of jitter between selection and outcome phases of each trial, allowing for disambiguation of all three phases.

Participants completed 66 trials, 33 choice and 33 no-choice. Trial order and the side of the screen on which the yellow and blue cards appeared were randomized, preventing final action planning. Decisions made by the participant during choice trials were mirrored by the computer with a three-trial lag during no-choice trials in an effort to match the overall rewards received across conditions of interest. Total task length was 14 minutes 59 seconds.

Participants were told their objective was to learn by trial and error which color card was more likely to give them points. They were informed that for some trials they would get to choose, but for others the computer would choose for them. During the latter trials, they had to follow the computer’s selection. Participants were also told that the reward contingencies remained consistent regardless of whether they or the computer were doing the choosing. A questionnaire administered after scanning asked participants to rate their desire to win points on a scale of 1 to 10 and their preference for choice or no-choice trials.

### Functional Magnetic Resonance Imaging Data Analysis

Two analytic approaches were adopted:

1. The basic model was used to (a) verify that appropriate reward responses were seen for the outcome phase contrast of reward 100 > 0, regardless of choice/no-choice; (b) verify the results previously reported by Leotti and Delgado (15) regarding cue phase choice > no-choice activation; (c) assess choice > no-choice activation during the reward phase and the choice × reward interaction; and (d) examine the associations between contrasts from (a) and (b) with the three General Causality Orientations Scale causality orientations (autonomy, control, impersonal), neuroticism, and depressive symptoms.
2. The Pavlovian reward learning model attempted use a computational framework to estimate how much each participant valued being able to choose by fitting a temporal difference learning model to the data. This considered the cue phase choice and no-choice indicators as though they were stimuli to be conditioned on subsequently obtained rewards. We proposed that the degree to which the model’s value estimate accounted for choice anticipatory activation would be dependent on causality orientation, neuroticism, and depressive symptoms.

### Basic Model

This was modeled at the first level as a series of delta functions convolved with a canonical hemodynamic response function, the onsets of which were denoted by experimental conditions of interest. These were the onsets of the choice and no-choice cues and the onsets of trial outcome, with choice/no-choice and 0/100 points being modeled separately, giving six experimental vectors of interest. Nuisance regressors included the onsets of yellow/blue selection, trials where an incorrect response or no response was received, and motion parameters.

At the second level, cue phase contrasts of choice > baseline and no-choice > baseline were entered into a random-effects flexible factorial analysis, modeling the factors of participant and choice/no-choice. The outcome phase was considered in a separate 2 × 2 flexible factorial analysis incorporating the contrasts of choice 100 > baseline, choice 0 > baseline, no-choice 100 > baseline, and no-choice 0 > baseline, modeling the factors of participant, choice/no-choice, and reward amount (see Supplement). For both models, each participant’s desire to win points and any difference in points received for choice versus no-choice trials were included as nuisance covariates. Regions identified as showing significant activation for the contrasts of interest were subject to extraction of the first eigenvariate for the suprathreshold cluster and their relationships with our covariates of interest explored (autonomy, control, impersonal, Quick Inventory of Depressive Symptomatology [QIDS] depressive symptoms, and Eysenck Personality Questionnaire Revised neuroticism scores). This was done using backward regression in IBM SPSS Version 23 (IBM Corp., Armonk, NY): for each extracted region, the model that best accounted for the data was identified by analysis of variance; within this, significant coefficients of explanatory covariates were reported. These were subjected to false discovery rate correction with *Q* = .05 across all comparisons, and standardized β values were reported.

### Pavlovian Reward Learning Model

The task was also modeled as an instance of classical conditioning, using a temporal difference learning model (21). We wished to identify if learning rate varied according to whether or not participants were actively choosing. The model implemented four different learning rates, 0.2, 0.4, 0.6, and 0.8, used to generate cue value and prediction error (PE) estimates across the task for each participant based on their cue-outcome experiences during the scan. The unconditioned stimulus was the outcome phase of each trial (the receipt of 100 or 0 points). The conditioned stimuli were the choice and no-choice indicators during the cue phase (see Supplement). Cue value was used to modulate trial-by-trial regressors representing the cue phase of each trial, and PE was used to modulate the outcome phase. Choice and no-choice conditions were modeled separately. These were entered into first-level SPM analyses, with a different SPM for each learning rate. Contrast estimates for each regressor were taken into second-level 2 × 4 flexible factorial analyses, which modeled the main effects of participant, choice/no-choice, and learning rate. As we expected choice value estimates to strongly covary with measures of autonomy, control, impersonal, neuroticism, and depression scores, these were included in the second-level analyses, modeling interactions with both choice/no-choice and learning rate.

For both the basic and Pavlovian models, second-level contrasts were evaluated at a whole-brain voxel height threshold of *p*_*familywise error–corrected*_ < .05. Given a priori interest in the striatum and dopaminergic midbrain, we also conducted region-of-interest analyses within a structurally defined mask comprising bilateral caudate, putamen, and dopaminergic midbrain (see Supplement). Masked voxels were reported as significantly activated if they exceeded a familywise error–corrected height threshold of *p* < .05.

## Results

### Demographics, Neuropsychology, and Symptoms

Median age of participants was 62 years, and 46% were men (Table 1). There was no correlation between age and task performance (*p* > .823). Of participants, 93% preferred making their own choices. Learning continued throughout the task, with the most rewarding card being chosen 79% of the time during the final quarter of the session (Supplemental Figure S3). Both QIDS depression (τ = .213, *p* = .003) and impersonal scores (τ = .197, *p* = .003) were positively correlated with neuroticism.

**Table 1:** Demographic, Personality, Symptom, and Behavior Measures

Measure (Possible Range)	Median (IQR)	Skewness	Kurtosis	Kendall’s τ Correlation (*p*) (Neuroticism)
Age, Years	62.0 (3.00)	−0.574	0.630	
Sex, F/M	56/66			
Handedness, R/L/A	111/5/6			
GCOS: Autonomy (12–84)	68 (10)	−0.881	1.071	
GCOS: Control (12–84)	48 (10)	−0.007	0.637	
GCOS: Impersonal (12–84)	37 (16)	−0.176	−0.714	.197 (.003)
Neuroticism (0–12)	2 (3)	1.217	1.843	
QIDS (0–27)	3 (2)	1.262	2.382	.213 (.003)
Desire to Win (1–10)	7.5 (4.0)	−0.742	−0.047	
Trial Preference, Choice/No-Choice	114/9			
Points Won (0–6600)	3500 (1200)	−0.384	0.017	
No-Choice/Choice Points	0.623 (0.39)	−0.044	−1.167	
No-Choice Trials Missed (0–33)	1.00 (3.00)	1.840	2.675	

Only significant correlations between personality and symptom measures are shown. No-choice trials where the participant chose the card not preselected by the computer were defined as missed. SE of skewness was 0.220 and of kurtosis was 0.437.

A, ambidextrous; F, female; GCOS, General Causality Orientations Scale; IQR, interquartile range; L, left; M, male; QIDS, Quick Inventory of Depressive Symptomatology; R, right.

### Basic Model: (a) Reward Verification

See Supplement.

### Basic Model: (b) Verifying Anticipation of Choice: Cue Phase

**Figure 2:**
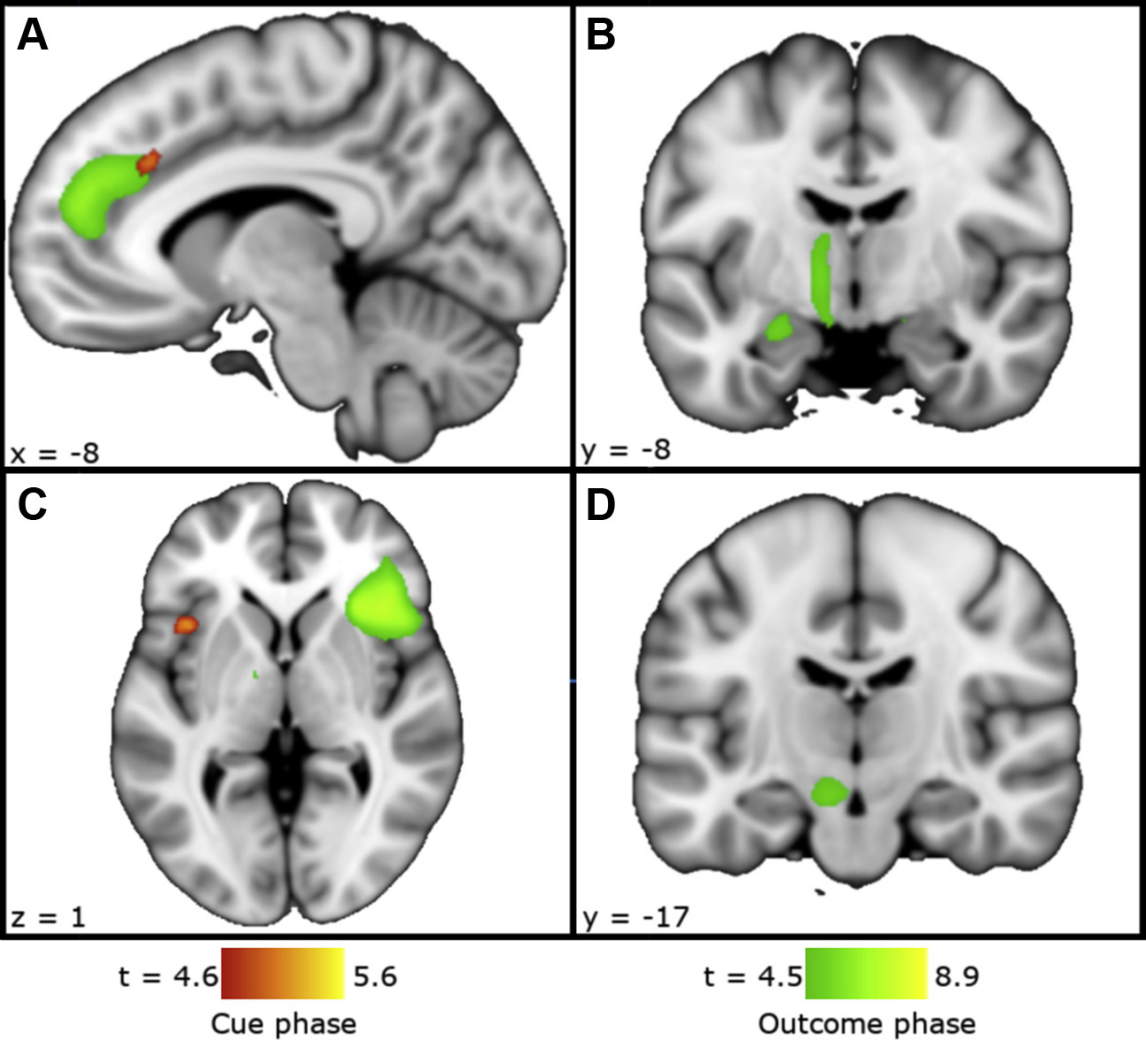
Choice > no-choice activation during the cue (red) and outcome (green) phases. **(A)** Left cingulate cortex. **(B)** Left hippocampus and thalamus. **(C)** Anterior insula and inferior frontal cortex. **(D)** Left substantia nigra. Images shown achieve a whole-brain voxel height significance threshold of *p*_*familywise error–corrected*_ < .05.

**Figure 3:**
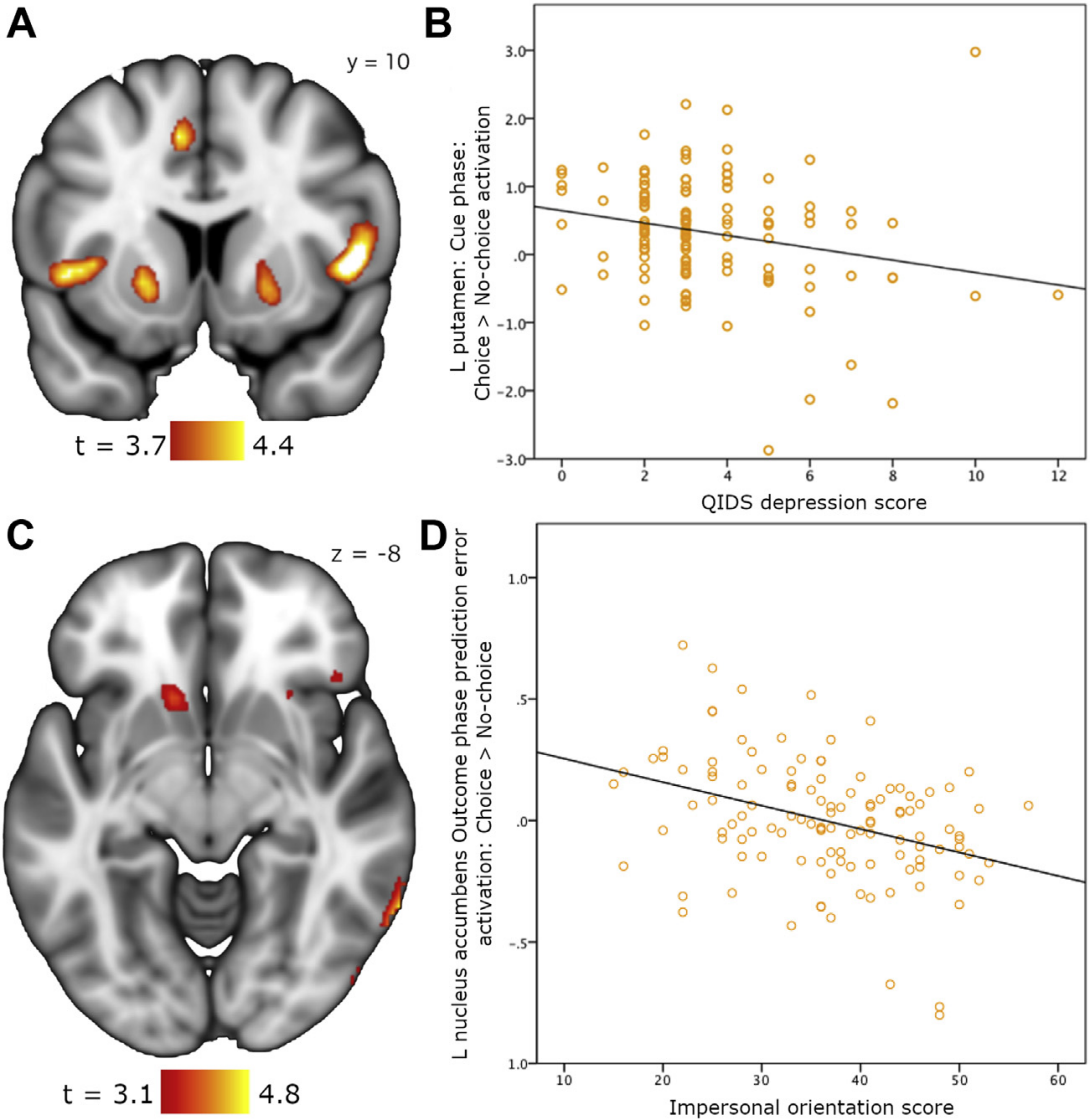
**(A)** Choice > no-choice anticipation (cue phase) contrast demonstrating activation in bilateral putamen. **(B)** Relationship between Quick Inventory of Depressive Symptomatology (QIDS) depression score and left (L) putamen activation (β = −.365, *p* < .001). **(C)** L nucleus accumbens shows a correlation between impersonal orientation and no-choice > choice effect for outcome phase prediction error encoding. **(D)** The relationship between impersonal orientation and L nucleus accumbens outcome phase prediction error activation. For panels **(A)** and **(C)**, results achieve a voxel-height significance of *p*_*familywise error–corrected*_ < .05 within the striatum/midbrain mask.

Cue phase choice > no-choice revealed strong activation in the cerebellum, left insula, left cingulate/supplementary motor area, and right inferior frontal gyrus (IFG), corrected for whole-brain volume (Figure 2 and Table 2). Bilateral putamen was activated within the striatum/midbrain a priori mask (Figure 3A). No-choice > choice showed activation in occipital cortex only (Table 2).

**Table 2:** Choice or No-Choice Anticipatory Activation During Cue Phase

Contrast	Region	MNI Coordinates			Voxels	*t*	*z*	*p*_*FWE-corrected*_
		x	y	z				
Choice > No-Choice	L cerebellum	−38	−56	−52	55	5.57	5.24	.001
	L insula	−40	16	2	63	5.29	5.01	.004
	R IFG	50	12	6	24	5.06	4.81	.010
	L cingulate/SMA	−10	28	32	20	5.04	4.80	.010
	R insula	40	18	6	5	4.68	4.48	.038
	L putamen	−20	10	−2	84	4.33	4.17	.008^a^
	R putamen	22	12	−4	55	4.19	4.04	.012^a^
No-Choice > Choice	R occipital cortex	12	−88	−2	118	6.56	6.05	< .001
	L occipital cortex	−18	−82	−14	148	6.11	5.69	< .001

FWE-corrected *p* values are for the whole-brain volume except where noted (see footnote below).

FWE, familywise error; IFG, inferior frontal gyrus; L, left; MNI, Montreal Neurological Institute; R, right; SMA, supplementary motor area.

a FWE-corrected significance within the striatum/midbrain mask.

### Basic Model: (c) Reward and Choice: Outcome Phase

Next we examined whether responses to personally earned outcomes differed from those passively received. The outcome phase choice > no-choice contrast showed significant activation in the bilateral insula, anterior cingulate, right IFG, left hippocampus, and left thalamus (Figure 2 and Table 3). Within the striatum/midbrain region of interest, there was significant choice > no-choice activation within the left substantia nigra and right caudate nucleus. No-choice > choice activated left middle frontal cortex, precuneus, and angular gyrus.

**Table 3:** Outcome Phase Activation: Choice Versus No-Choice

Contrast	Region	MNI Coordinates			Voxels	*t*	*z*	*p*_*FWE-corrected*_
		x	y	z				
Choice > No-Choice	R insula/IFG	36	20	−8	1789	8.93	Inf	< .001
	R cingulate/medial superior frontal cortex	0	38	28	3125	7.78	7.47	< .001
	L insula	−36	20	−10	303	6.05	5.90	< .001
	L ventral anterior thalamus	−12	−2	−6	34	4.69	4.62	.024
	L hippocampus	−26	−6	−16	4	4.60	4.53	.034
	L substantia nigra	−8	−14	−14	122	4.45	4.38	.003^a^
	R caudate	12	4	8	4	3.85	3.81	.030^a^
No-Choice > Choice	L MFG	−30	30	52	75	5.40	5.29	.001
	L precuneus	−2	−64	32	36	4.81	4.73	.015
	L angular gyrus	−48	−62	24	27	4.80	4.72	.015
Choice (0 > 100) > No-Choice (0 > 100)	R IFG	34	20	10	5	4.56	4.50	.038

FWE-corrected *p* values are for the whole-brain volume except where noted (see footnote below).

FWE, familywise error; IFG, inferior frontal gyrus; Inf, infinite; L, left; MFG, middle frontal gyrus; MNI, Montreal Neurological Institute; R, right.

a FWE-corrected significance within the striatum/midbrain mask.

### Reward × Choice Interaction Activations

**Figure 4:**
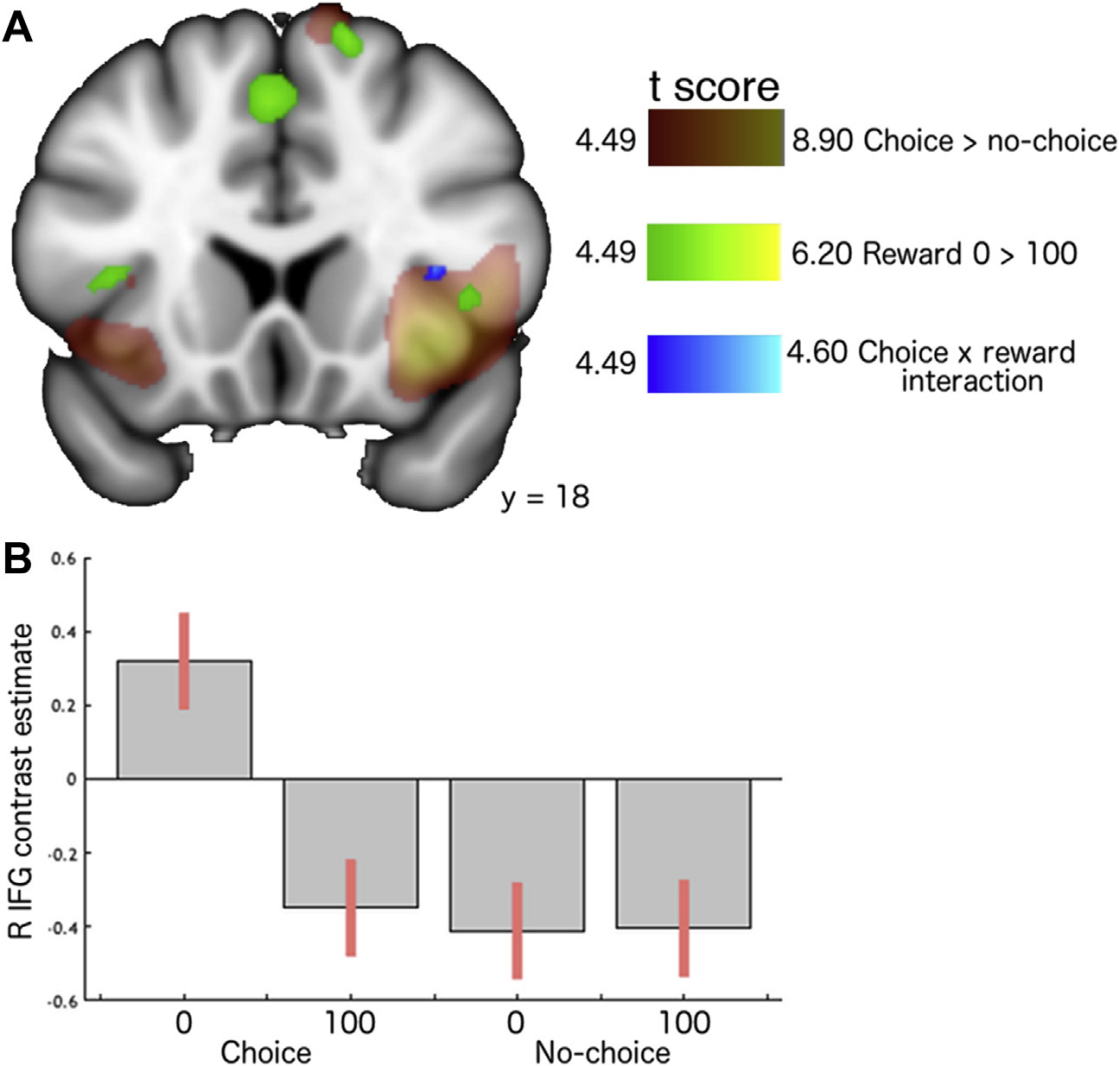
Choice × reward interaction within right (R) inferior frontal gyrus (IFG) during the outcome phase. **(A)** Activation maps for choice > no-choice (red), reward 0 > 100 (green), and choice × reward interaction (blue), displayed at a whole-brain voxelwise familywise error–corrected threshold of *p* < .05. **(B)** Contrast estimates extracted from the interaction cluster.

The contrast of choice (0 > 100) > no-choice (0 > 100) showed activation in right IFG pars opercularis (Table 3). Conjunction analysis confirmed that this lay within the choice > no-choice cluster (*p*_*familywise error–corrected*_ = .038) but not that of 0 > 100 (Figure 4A). Contrast estimates suggested enhanced activation when one personally failed to win (Figure 4B).

### Relations to Traits of Interest

QIDS depressive symptoms were negatively related to left putamen anticipation: choice > no-choice (β = −.365, *p* < .001) (Figure 3B). During the outcome phase, autonomy had a positive association with right IFG and insula choice > no-choice activation (β = .280, *p* = .045), whereas impersonal demonstrated the inverse relationship (β = −.255, *p* = .025). Within precuneus, control had a positive relationship during outcome: no-choice > choice activation (β = .396, *p* < .001). Conversely, impersonal showed a negative association in the same region (β = −.288, *p* = .012) (Supplemental Figure S4). Here QIDS depression demonstrated a similar pattern to control (β = .226, *p* = .039). Table 4 details these relationships.

**Table 4:** Relationships Between Significant Activation Clusters and Metrics of Interest (GCOS Causality Orientation, Neuroticism, and QIDS Depressive Symptoms)

Phase	Region	Contrast	Metric	Standardized β	*t (df)*	*p*_*corrected*_
Cue	L putamen	Choice > no-choice	QIDS	−.365	3.734 (113)	< .001
Outcome	R IFG/insula	Choice > no-choice	Autonomy	.280	2.253 (113)	.045
			Impersonal	−.255	2.758 (113)	.025
	L precuneus	No-choice > choice	Control	.396	4.888 (114)	< .001
			Impersonal	−.288	2.986 (115)	.012
			QIDS	.226	2.467 (113)	.039

GCOS, General Causality Orientations Scale; IFG, inferior frontal gyrus; L, left; QIDS, Quick Inventory of Depressive Symptomatology; R, right.

### Pavlovian Reward Learning: Value of Personal Choice

The final analytical thread considered whether the ability to choose was intrinsically rewarding in itself, within a reinforcement learning context. During the cue phase of each trial, there were no main effects of choice/no-choice or learning rate. However, as anticipated, there was significant covariation with several metrics of interest (Table 5). Increasing autonomy was associated with greater no-choice > choice value estimates in right amygdala (*p* = .008) and greater choice > no-choice value estimates in anterior caudate (*p* = .019). Again during the cue phase, control orientation demonstrated a positive relationship with learning rate in the right superior temporal sulcus (*p* = .017).

**Table 5:** Pavlovian Conditioning of Choice Versus No-Choice

Phase	Contrast	Region	MNI Coordinates			Voxels	*t*	*z*	*p*_*FWE-corrected*_
			x	y	z				
Cue (CS) × Model Value	Autonomy × (no-choice > choice)	R basolateral amygdala	26	0	−24	14	4.72	4.69	.008
	Autonomy × (choice > no-choice)	L dorsal anterior caudate	−18	18	8	20	4.49	4.46	.019
	Autonomy × decreasing α	L anterior caudate	−18	26	4	32	4.72	4.70	.003
	Control × increasing α	R superior temporal sulcus	48	−24	−6	31	4.52	4.49	.017
Outcome (US) × Model Prediction Error	Main effect: decreasing α	B ventral striatum	−12	10	−12	51	5.81	5.76	< .001
	Main effect: increasing α	R insula	−34	20	8	37	5.09	5.05	.005
	Main effect: increasing α	L supplementary motor area	−4	22	44	132	5.29	5.24	.002
	Impersonal × (no-choice > choice)	B nucleus accumbens	−12	16	−10	23	4.44	4.41	.004^a^

Multiplication sign (×) denotes an interaction between a General Causality Orientations Scale subscale and the contrast described. FWE-corrected *p* values are for the whole-brain volume except where noted (see footnote below).

B, bilateral; CS, conditioned stimulus; FWE, familywise error; L, left; MNI, Montreal Neurological Institute; R, right; US, unconditioned stimulus.

a FWE-corrected significance within the striatum/midbrain mask.

During the outcome phase, there was a significant main effect of learning rate (α) in ventral striatum, with a lower α being associated with greater PE representation (*p* < .001). Conversely, there was an effect of increasing α in the right anterior insula and supplementary motor area (*p* < .006). Learning in ventral striatum therefore appears to operate over a longer timescale than in insula and supplementary motor area. Finally, impersonal showed a stronger PE representation for no-choice > choice in bilateral nucleus accumbens (*p* < .004) (Figure 3C, D). For interest, results significant at *p* < .001 uncorrected can be found in the Supplement.

## Discussion

In this study, we modified Leotti and Delgado’s 2011 inherent reward of choice task (15) to 1) verify their previous findings, 2) disambiguate the cue and outcome phases, 3) demonstrate the utility of computational models in this context, and 4) see whether task-elicited activation covaried with personality factors of relevance to depression. Their findings concerning choice anticipation were replicated within our larger independent sample of healthy control subjects. The task was amenable to Pavlovian reward learning analysis. We then demonstrated a series of novel findings within regions key to reward and depression, their relationship to depressive symptoms, and measures that attempt to personalize notions of reward and value. This aligns them with the depressive phenomena of motivational anhedonia and devaluation of the self.

### Anticipating Choice

We verified the striatal anticipatory response to choice as seen in Leotti and Delgado (15). Critically, we observed that this effect was diminished in participants with more depressive symptoms, suggesting an impairment in the hedonic value or salience attributed to personal choice. Reduced ventral striatal reward-linked responses are a well-replicated finding in patients with MDD, be it when viewing positive images (22), which correlates with anhedonia (23), or anticipating and receiving rewarding outcomes 24, 25. In healthy control subjects, depressive symptoms correlate with a reduction in the usual performance-enhancing effects of positive feedback, implying striatal dysfunction (26). Striatal activation correlates with enhanced recall of personally chosen items and exerts a modulatory effect over hippocampus (27): this mechanism may underpin the cognitive biases observed in MDD. It is notable that we too report striatal dysfunction in a group of healthy control participants, who have not been subject to the effects of medication or an episodic illness, while having a narrower distribution of depressive symptoms. We also found enhanced insula and cingulate activation during choice > no-choice anticipation: these regions have been shown to correlate with momentary subjective well-being in rewarding contexts (28), supporting the view that personal choice is intrinsically appetitive. Both are key components of the salience network and play a role in cognitive control (29).

### Personally Earned Versus Passively Received Rewards

Responses to personally chosen outcomes were enhanced compared with those that were passively received: insula/IFG and cingulate cortex were apparent, as were hippocampus, thalamus, and substantia nigra. Right IFG pars opercularis demonstrated a choice × reward interaction, whereby there was an enhanced response when participants personally failed to win. Right IFG pars opercularis plays a specific role (30) in the inhibition of motor and affective responses (31). It is also activated by personal regret versus simple disappointment (32). It could be argued that personally failing to win induces a self-blame response (33) that requires inhibition or emotional regulation. Such a response would be relevant to depression and particularly to resilience in the face of adversity (34).

### Inferior Frontal Gyrus and Goal-Sensitive Self-Regulation

Right IFG and insula showed a choice > no-choice response across the sample during the outcome phase, which was enhanced by high autonomy but diminished by high impersonal scores. The concept of locus of causality is not far removed from that of learned helplessness, which inspired an animal model of MDD and gave ventral prefrontal cortex (IFG) particular prominence in a recent update by its architects Maier and Seligman (35). Prolonged aversive events are proposed to stimulate the raphe nuclei, releasing serotonin within the striatum (inhibiting behavior) and amygdala (inducing fear and anxiety), regardless of detected contingencies. This response is inhibited if the agent has previous experience of acting to escape aversive events, mediated by the regulatory influence of ventral prefrontal cortex over the raphe nuclei and striatum. Maier and Seligman suggest that this process equates to the agent’s being able to imagine having control over future aversive situations. Right IFG and insula are crucial contributors to cognitive control, governing the ability to select and maintain goal-directed action at the expense of other alternatives (36). Strong evidence from a meta-analysis supports their role in the cognitive reappraisal of emotional stimuli (37). Reduced responses to negative affective stimuli have been reliably demonstrated in patients with MDD (38). In this study, we show the response of IFG to personal choice is greater in individuals having high autonomy and reduced in individuals having an impersonal, passive style. The latter may therefore have a reduced ability to act to escape aversive situations and regulate subcortical limbic responses to aversive events, whereas the former would be more adaptive and resilient. Bhanji *et al.* (39) linked resilience to believing one has personal control: they found that one’s ability to overcome setbacks was reduced following exposure to an acute stressor; however, this was diminished in people who believed that they had some control over the setbacks.

### Precuneus and Agency Perception

The precuneus showed a no-choice > choice response during the outcome phase, especially so in participants with high control, with the opposite being seen with high impersonal scores. Precuneus is part of the default mode network and generally deactivates during goal-directed tasks (40). This happens to a lesser degree during tasks having a self-referential component, taking a first-person perspective, or inducing the experience of agency (41). It also activates when mentally simulating the actions of another versus oneself (42), taking perspectives alternative to one’s own (43), and considering the emotional states of both oneself and others versus neutral judgments (44). More abstractly, it is activated during judgments of intentional versus simple physical causality (45). In summary, it is arguable that any process that involves consideration of an intentional agent engages precuneus, regardless of whether this is one’s own self, although the self is likely to prevail during default mode operations. The control orientation may increase the propensity to seek cues in the minds of others and consider the computer’s intentions. Conversely, the impersonal orientation shows an apparent abolition of the effect seen in the general sample, suggesting a reduced inclination to consider intentionality at all.

### Reinforcement Learning

The final analysis phase attempted to capture the learning process underlying how the choice/no-choice cues developed their inherently rewarding character and how this related to participants’ characteristics. The use of a computational model potentially allows for a more mechanistic understanding of the activation observed and highlighted relationships with personality metrics that were not detected during the basic analysis. Highly autonomous people encoded value during presentation of the no-choice cue within right amygdala, suggesting that either no-choice cues (46) or the uncertainty associated with what the computer might select (47) was regarded as aversive. They also showed greater choice > no-choice cue valuations in dorsal anterior caudate, which through its interactions with prefrontal cortex plays a crucial role in goal-directed action (48). High control participants showed enhanced learning in the right superior temporal sulcus, which is especially involved in considering the intentions of external others (49). Finally, high impersonal participants had stronger nucleus accumbens PE signals for passively received rewards, suggesting that a reduced belief in the ability to control one’s behavior related to more reward system reactivity to gifted versus earned rewards.

### Limitations

A number of study participants were unable to perform the task correctly, suggesting that it was subjectively hard to understand or that a potentially important section of the population was excluded. We have not examined trial-by-trial assessments of choice preference or changes in stay/switch behavior, which are also believed to covary with depressive symptoms (26). The 80:20 yellow:blue reward contingency was used to permit reliable learning across a range of participants and may have induced ceiling effects in some participants, as we did not find a simple interaction between choice/no-choice and learning rate. However, it allowed us to focus on whether or not the participant did the choosing, without that choice in itself being particularly onerous. Indeed, additional confounds may have been introduced if there was a difference in decision-related deliberation between the choice and no-choice conditions. Alternatively, our temporal difference learning model may not have adequately captured the variance introduced by personal choice.

### Clinical Relevance

Our findings suggest that the modified inherent value of choice task could provide useful insights into the neurobiology of MDD. Within this large sample of healthy control subjects, we have shown how personal choice modulates activation within areas known to be disrupted in MDD. This covaries with how inclined participants are to see themselves as drivers of their actions, to look to the outside world for their cues, or even to feel at a loss as to why they act at all. Being able to tease apart how particular manifestations of personality impact one’s vulnerability to MDD is likely to be important to stratification. Characteristics such as causality orientation arguably build on more stable and heritable measures such as neuroticism, as they are more responsive to environmental events and so may provide more timely information regarding the risk of transition to illness as well as offering targets for psychotherapeutic interventions. The hope is that by examining the reward system in a manner that ties self-perception to behavior, more clinically applicable insights can be drawn. For example, a particularly effective therapeutic strategy for individuals having a high impersonal/low autonomy style might be to both enhance dopaminergic transmission and challenge self-orientation beliefs during cognitive behavioral therapy.
