# Supplementary material for: The Neurobiology of Personal Control During Reward Learning and Its Relationship to Mood
Source: Biol Psychiatry Cogn Neurosci Neuroimaging. 2019 Feb;4(2):190–9. doi: 10.1016/j.bpsc.2018.09.015 (PMC6374985; doi:10.1016/j.bpsc.2018.09.015)
Supplement: Supplemental Material [file mmc1.pdf]

The Neurobiology of Personal Control During Reward Learning and Its Relation to Mood

Supplemental Information

Neuropsychology and behavioural analyses

The General Causality Orientations Scale (GCOS), is derived from Self-Determination Theory (1), examines the sources from which a person is motivated to act (2), consisting of 3 dimensions: Autonomy, Control and Impersonal. Participants rate 12 vignettes of situations probing these dimensions, with total subscale scores ranging between 12 and 84. Neuroticism scores were derived from the Eysenck's Personality Questionnaire – Revised (EPQ-R; short form (3)). The severity of depressive symptoms were derived by self-report using the Quick Inventory of Depressive Symptomology (QIDS; (4)). Handedness was determined using the Edinburgh Handedness Inventory (5).

Neuroimaging data acquisition and preprocessing

Data was acquired using a Philips Achieva 3T TX-series scanner (Philips Healthcare, Best, Netherlands) at the University of Aberdeen, with a 32-channel phased-array head coil with a back-facing mirror (software version 5.1.7; gradients with maximum amplitude 80 mT/m and maximum slew rate 100 T/m/s). A projector and "Presentation" (Neurobehavioural Systems) version 18.1 were used for the presentation of task based fMRI.

fMRI data were acquired with a TR = 1.56s and TE = 26ms. FA = 70', FOV = 217mm, matrix size = 64 x 64. In-plane resolution was 3.4 x 3.4mm, with 32 5mm axial slices being acquired continuously with no gap. 573 volumes were collected, with the first six being discarded to

accommodate T1 saturation effects. A T1-weighted structural image was acquired as 160 sagittal slices, with TR = 8.3ms, TE = 3.8ms, TI = 1031ms, FA = 8', FOV = 240mm, matrix size = 240 x 240, giving a resolution of 0.9 x 0.9 x 1.0mm. Data were preprocessed and analysed using SPM12 (Wellcome Department of Imaging Neuroscience, London, England; http://www.fil.ion.ucl.ac.uk/spm/software/spm12/), within MathWorks MATLAB R2016a (http://www.mathworks.com). fMRI volumes were reconstructed into NIfTI format, and realigned to the mean volume. The structural image was segmented and warped to MNI space. The mean fMRI and structural volumes were coregistered, and the normalisation parameters applied to the whole fMRI dataset which was then smoothed using an 8mm FWHM gaussian kernel, and resampled at 2mm isotropic resolution. The data was high pass filtered with 128s cutoff, and serial correlations modelled using a first-order autoregressive model.

Basic model flexible factorial analysis

For both the cue phase and outcome phase second level analyses, the factor of participant was modelled as having independent and equal variance, as it was not anticipated that the healthy control population from which the sample was drawn would demonstrate wide variance between individuals. The factor of Choice/No-choice was also modelled for both these analyses, this time having dependent and equal variance, as this was a within-subjects comparison. The outcome phase analysis included the additional factor of reward amount (100 or 0 point): this was again modelled as dependent and equal variance, for the same reasons as Choice/No-choice.

Pavlovian reward learning methods

Each trial was modeled over six time points, with the CS occurring at t = 1, and US at t = 3. The predicted value for each CS was calculated at each time point:

$$\hat{V}(t) = wx(t)$$

Where V, w and x are vectors with separate entries for the Choice and No-choice indicators. X(t) is a binary vector denoting the presence of each Choice/No-choice indicator and w is the learned weight accorded to each CS. The predicted value V is updated at each time step according the PE, that is, to the difference between its estimation at the current time step, and the next:

$$\delta(t) = r(t) + \lambda \hat{V}(t+1) - \hat{V}(t)$$

Where r(t) is the outcome at time t. This is 1 when 100 points are received, and 0 otherwise. λ is a temporal discounting factor, which was set to 1. Weights were updated for each trial according to:

$$\Delta w = \alpha \sum_t x(t)\delta(t)$$

Where α is the learning rate.

Functional imaging mask

The striatal/dopaminergic midbrain mask was created from a union of Automated Anatomical Labeling-defined caudate and putamen; Brodmann-defined substantia nigra; and a 10mm sphere centred on the ventral tegmental area at MNI coordinates 0 -20 -10 (6), using the WFU Pickatlas (7; 8).

Behavioural results

For a number of our participants, it was found that a random run of No-choice trials would lead to there being a relatively insufficient number of Choice trials for the computer to copy the behaviour of. In these circumstances, the system would default to forcing the participant to select the losing card during No-Choice trials. This led to some of our participants receiving a lower number of points for No-choice relative to Choice trials (Supplemental Figure S1). This issue had no correlation with age, QIDS depression, neuroticism or causality orientation metrics ($p > 0.208$). Point difference was included as a nuisance regressor in all the imaging analyses described here. We also performed an additional series of analyses where an additional factor of Group was modelled whereby participants were divided into independent groups with No-Choice/Choice scores <= 0.4, 0.4 < scores <= 0.6, 0.6 < scores <= 0.8 and scores > 0.8. The interaction between score group and Choice/No-choice was examined. Contrast estimates were also extracted to demonstrate how activation varied according to Choice and score group.

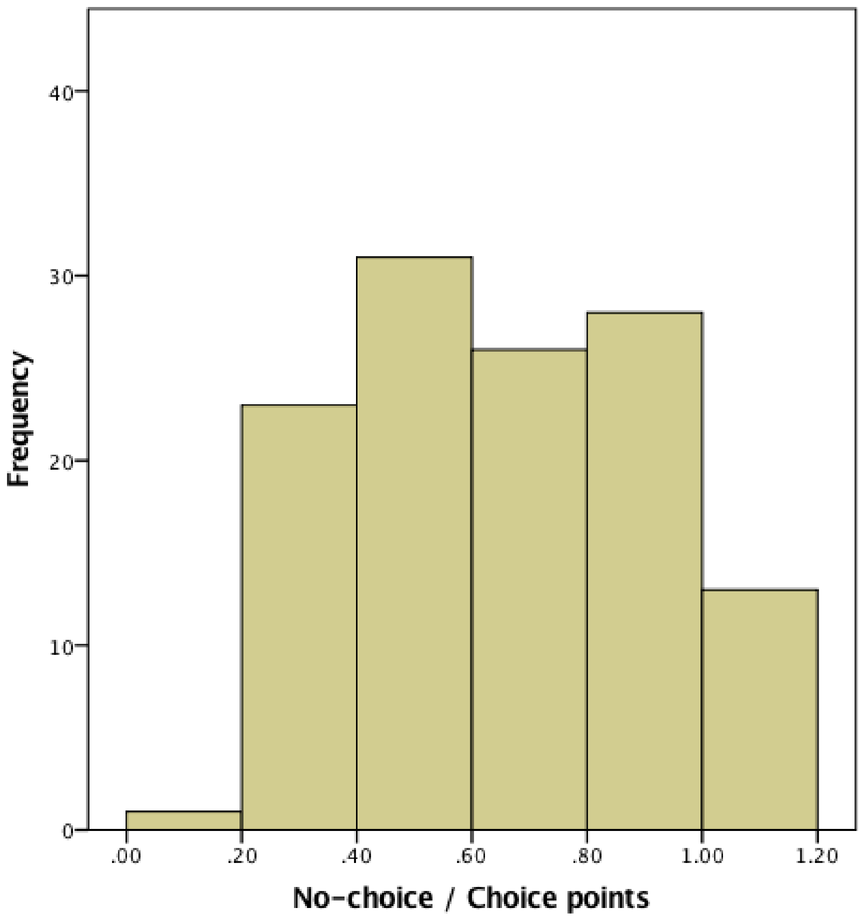

Supplemental Figure S1: The distribution of the difference in points for Choice and No-choice trials.

During the Cue phase for the basic analysis (analogous to Table 1 in the main paper), there were no significant interactions between Choice/No-choice and score group ($P > 0.246$). Representative extracted contrast estimates for left insula and right putamen are shown in Supplemental Figure S2(a), which suggest that the Choice > No-choice effects reported are not attributable solely to point differences.

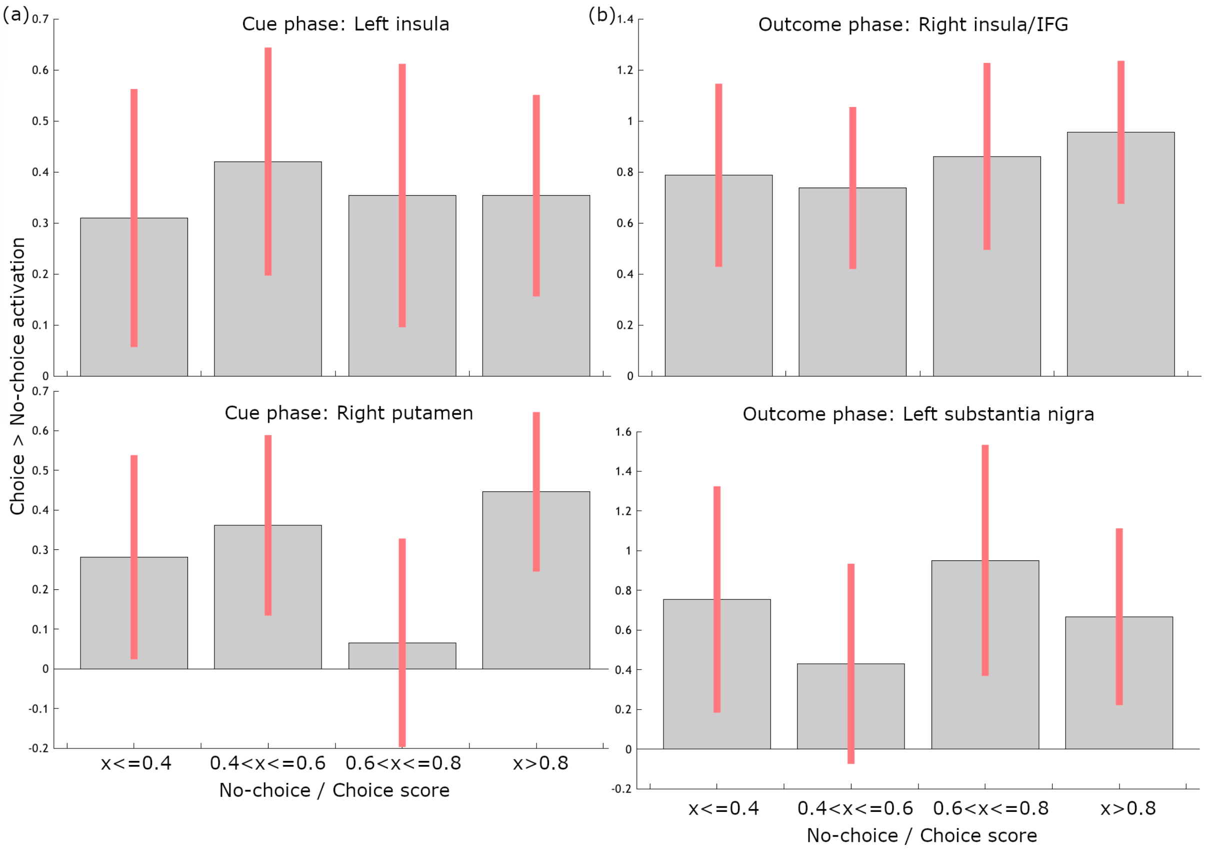

Supplemental Figure S2: (a) Cue phase contrast estimates demonstrating the apparent lack of impact of point differences on Choice > No-choice activation in two key regions reported in Table 2. (b) Outcome phase contrast estimates for two regions reported in Table 3.

Likewise, for the Outcome phase, there were no score group x Choice/No-choice interactions ($p > 0.432$). Supplemental Figure S2(b) provides representative contrast estimates.

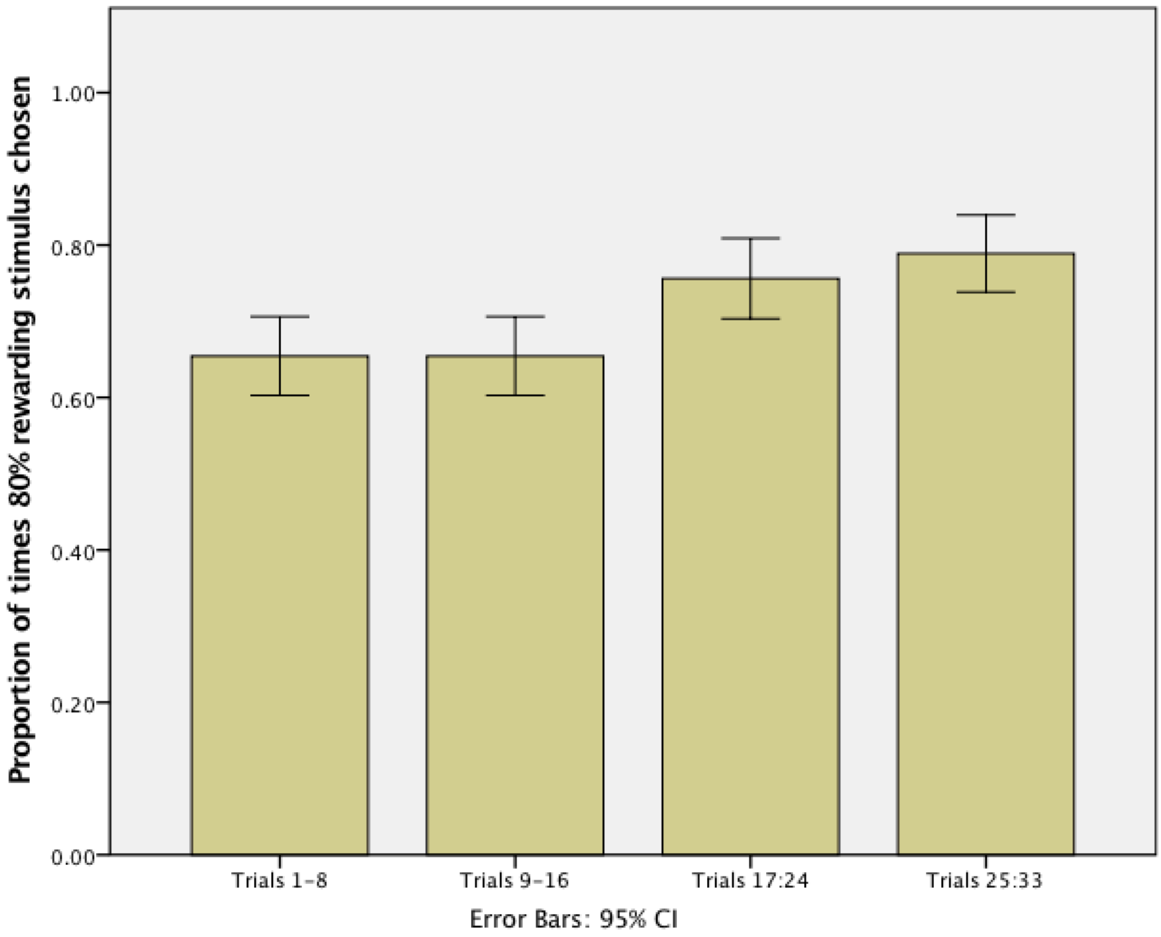

Supplemental Figure S3: Proportion of times the yellow (most rewarding) card was chosen during Choice trials.

Basic model results: Outcome phase, reward 100 <> 0

The main effect of reward 100 > 0 across both the Choice and No-choice conditions revealed robust activation within regions previously associated with typical reward responses, such as the nucleus accumbens and orbitofrontal cortex. The inverse contrast of 0 > 100 showed right angular gyrus, left supplementary motor area (SMA) and bilateral insula/inferior frontal gyrus (IFG) activation (Supplementary Table S1).

Supplemental Table S1: Outcome phase activation: reward 100 versus reward 0.

Contrast	Region	MNI coords	Voxels	T	Z	P (FWE-corrected)
Reward 100 > 0	L + R occipital cortex	16 -94 10<br>-14 -96 0	13832	29.17	Inf	<0.001
	L precentral gyrus	-52 0 50	223	7.75	7.45	<0.001
	L nucleus accumbens	-10 8 -12	102	6.42	6.24	<0.001
	L STG	-56 -6 -12	200	6.42	6.24	<0.001
	R medial SFG	6 54 -8	504	5.56	5.44	0.001
	R ventral putamen	14 8 -8	4	5.13	5.04	0.004
	L orbitofrontal cortex	-24 32 -16	16	4.72	4.65	0.021
Reward 0 > 100	R angular gyrus	50 -56 56	998	6.15	5.99	<0.001
	L SMA	-4 22 46	252	5.30	5.20	0.002
	R SFG	16 18 62	53	5.04	4.95	0.006
	L insula/IFG	-38 20 -8	25	4.90	4.82	0.010
	R insula/IFG	44 18 4	12	4.76	4.69	0.018

FWE-corrected p values are for the whole brain volume. IFG: inferior frontal gyrus. SFG: superior frontal gyrus. SMA: supplementary motor area. STG: superior temporal gyrus.

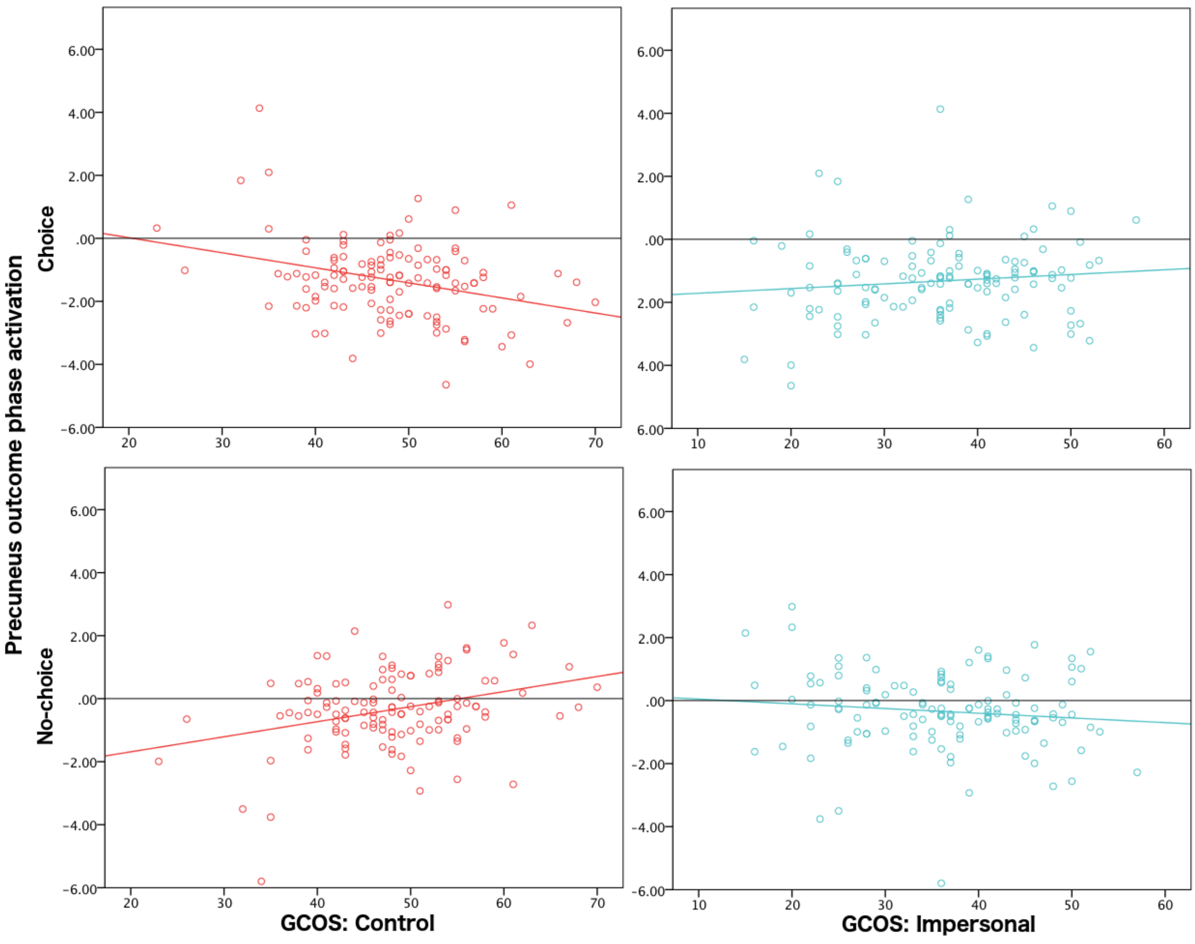

Supplemental Figure S4: Precuneus outcome activation in relation to Control (β = 0.396, p < 0.001) and Impersonal scores (β = -0.288, p = 0.012).

Supplemental References

1. Ryan RM, Deci EL (2017): *Self-Determination Theory*. Guilford Publications.

2. Deci EL, Ryan RM (1985): The general causality orientations scale: Self-determination in personality. *Journal of Research in Personality*. 19: 109–134.

3. Eysenck HJ, Eysenck SBG (1994): *Manual for the Eysenck Personality Questionnaire*.

4. Rush AJ, Giles DE, Schlesser MA, Fulton CL, Weissenburger J, Burns C (1986): The Inventory for Depressive Symptomatology (IDS): preliminary findings. *Psychiatry Res*. 18: 65–87.

5. Oldfield RC (1971): The assessment and analysis of handedness: the Edinburgh inventory. *Neuropsychologia*. 9: 97–113.

6. Romaniuk L, Honey GD, King JRL, Whalley HC, McIntosh AM, Levita L, *et al.* (2010): Midbrain activation during Pavlovian conditioning and delusional symptoms in schizophrenia. *Arch Gen Psychiatry*. 67: 1246–1254.

7. Lancaster JL, Woldorff MG, Parsons LM, Liotti M, Freitas CS, Rainey L, *et al.* (2000): Automated Talairach atlas labels for functional brain mapping. *Hum Brain Mapp*. 10: 120–131.

8. Maldjian JA, Laurienti PJ, Kraft RA, Burdette JH (2003): An automated method for neuroanatomic and cytoarchitectonic atlas-based interrogation of fMRI data sets. *NeuroImage*. 19: 1233–1239.
